# Characterization of a novel *N*-acetylneuraminic acid lyase favoring *N*-acetylneuraminic acid synthesis

**DOI:** 10.1038/srep09341

**Published:** 2015-03-23

**Authors:** Wenyan Ji, Wujin Sun, Jinmei Feng, Tianshun Song, Dalu Zhang, Pingkai Ouyang, Zhen Gu, Jingjing Xie

**Affiliations:** 1 State Key Laboratory of Materials-Oriented Chemical Engineering, Nanjing, PR China; 2 College of Life Science and Pharmaceutical Engineering, Nanjing Tech University, Nanjing, PR China; 3 National Engineering Technique Research Center for Biotechnology, Nanjing, PR China; 4 Joint Department of Biomedical Engineering, University of North Carolina at Chapel Hill and North Carolina State University, Raleigh, United States; 5 Department of Pathogenic Biology, School of Medicine, Jianghan University, Wuhan, China; 6 International Cooperation Division, China National Center for Biotechnology Development, Beijing, PR China

## Abstract

*N*-Acetylneuraminic acid lyase (NAL, E.C. number 4.1.3.3) is a Class I aldolase that catalyzes the reversible aldol cleavage of *N*-acetylneuraminic acid (Neu5Ac) from pyruvate and *N*-acetyl-D-mannosamine (ManNAc). Due to the equilibrium favoring Neu5Ac cleavage, the enzyme catalyzes the rate-limiting step of two biocatalytic reactions producing Neu5Ac in industry. We report the biochemical characterization of a novel NAL from a “GRAS” (General recognized as safe) strain *C. glutamicum* ATCC 13032 (CgNal). Compared to all previously reported NALs, CgNal exhibited the lowest *k*_cat_/K_m_ value for Neu5Ac and highest *k*_cat_/K_m_ values for ManNAc and pyruvate, which makes CgNal favor Neu5Ac synthesis the most. The recombinant CgNal reached the highest expression level (480 mg/L culture), and the highest reported yield of Neu5Ac was achieved (194 g/L, 0.63 M). All these unique properties make CgNal a promising biocatalyst for industrial Neu5Ac biosynthesis. Additionally, although showing the best Neu5Ac synthesis activity among the NAL family, CgNal is more related to dihydrodipicolinate synthase (DHDPS) by phylogenetic analysis. The activities of CgNal towards both NAL's and DHDPS' substrates are fairly high, which indicates CgNal a bi-functional enzyme. The sequence analysis suggests that CgNal might have adopted a unique set of residues for substrates recognition.

Sialic acids are a family of 9-carbon amino sugars involved in the modulation of various biological processes^1^. Among more than 50 structurally distinct sialic acids that have been found in nature, *N*-acetylneuraminic acid (Neu5Ac) is the most ubiquitous form^2^,^3^. Neu5Ac has great potential in pharmaceutical and food industry: Neu5Ac is a potential raw material in the synthesis of anti-influenza drugs to prevent both influenza types A and B infections^4^ and it is also an important additive in dairy products for its ability to strengthen the immunity of infants^5^.

*N*-acetylneuraminic acid lyase (NAL, EC 4.1.3.3) has been employed in industrial production of Neu5Ac^1^,^6^,^7^ and it catalyzes the reversible aldol condensation of Neu5Ac from *N*-acetyl-D-mannosamine (ManNAc) and pyruvate (Fig. 1a). NAL is ubiquitously distributed in nature, such as bacteria^8^ and mammals (human^9^, beef^10^, pig^11^). Pathogenic bacteria colonized human respiratory tract and gut to utilize sialic acid as carbon and nitrogen source^8^ and disruption of the NAL gene could severely reduce the virulence of *Vibrio vulnificus*^12^. In addition to pathogens, NAL was also reported from “generally regarded as safe” (GRAS) organisms, such as *Lactobacillus plantarum* WCFS1^13^ and *Taphylococcus carnosus* TM300^14^.

In order to understand its function and reaction mechanism, crystal structures of *E. coli* NAL (EcNal, PDB ID: 1NAL, 1FDY, 1FDZ and 1HL2)^15^,^16^,^17^, *Haemophilus influenzae* NAL (PDB ID: 1F5Z)^18^ and *Pasteurella multocida* NAL (PDB ID: 4IMC, 4IMD, 4IME, 4IMF and 4IMG)^19^ were solved and studied. NAL belongs to aldolase Class-I superfamily, which has a classic (α/β)_8_ barrel profile, and activated as a homotetramer^8^,^20^,^21^. The NAL family shares a unifying mechanism as showing in EcNal. The catalytic Lys165 of EcNal forms schiff base with α-keto moiety of pyruvate, and the highly conserved Tyr137 is associated with aldol cleavage/condensation step^18^. Motif GXXGE (Gly46-Glu50 in EcNal), and the residues Asp191, Glu192 and Ser208 contribute to substrate recognition^14^,^18^. Other members of the superfamily include dihydrodipicolinate synthase (DHDPS), D-5-keto-4-deoxyglucarate dehydratase (KDGDH), *trans*-o-hydroxybenzylidenepyruvate hydrolase-aldolase (HBPHA), *trans*-2′-carboxybenzalpyruvate hydratase-aldolase (CBPHA) and 2-keto-3-deoxygluconate aldolase (KDGA)^18^,^22^,^23^,^24^. Among them, DHDPS (EC 4.3.3.7) is a member that shows the highest similarity with NAL. It catalyzes the first step in lysine biosynthesis and condensed L-aspartate-β-semialdehyde (ASA) with pyruvate to synthesize dihydrodipicolinate (Fig. 1b)^15^ The homology of Nal and DHDPS has been evidenced by a single mutation in *E. coli* NAL (L142R) to shift NAL's activity towards DHDPS^17^.

As an important biocatalyst for *in vitro* chemoenzymatic synthesis of Neu5Ac and its derivatives, recombinant NAL has been broadly applied with either *E. coli*^25^ or plants^26^ as host. While naturally, NAL primarily functions to regulate intercellular sialic acid metabolism in mammalian cells; some microorganisms use NAL to catabolize sialic acid for a carbon and energy source^19^. Through a long process of evolution, natural selection is more inclined to have a NAL with relatively high Neu5Ac cleavage ability. Therefore, all reported NAL to date suffered a common drawback: The equilibrium of the reversible reaction favors Neu5Ac cleavage rather than synthesis^11^,^13^,^14^,^27^,^28^,^29^, which might lead to low yield and low efficiency for industrial production of Neu5Ac.

In this study, we report a novel NAL (CgNal) from a “GRAS” organism *Corynebacterium glutamicum* ATCC 13032, which shows dual functions as NAL and DHDPS. More importantly, as NAL, CgNal shows a unique property of favoring Neu5Ac synthesis in the reversible reaction. In addition, the recombinant CgNal obtained a high expression level and achieved high production yield of Neu5Ac. These characteristics make CgNal a promising biocatalyst in industrial Neu5Ac biosynthesis processes.

## Results

### Cloning, expression and purification of CgNal

**Figure 2:**
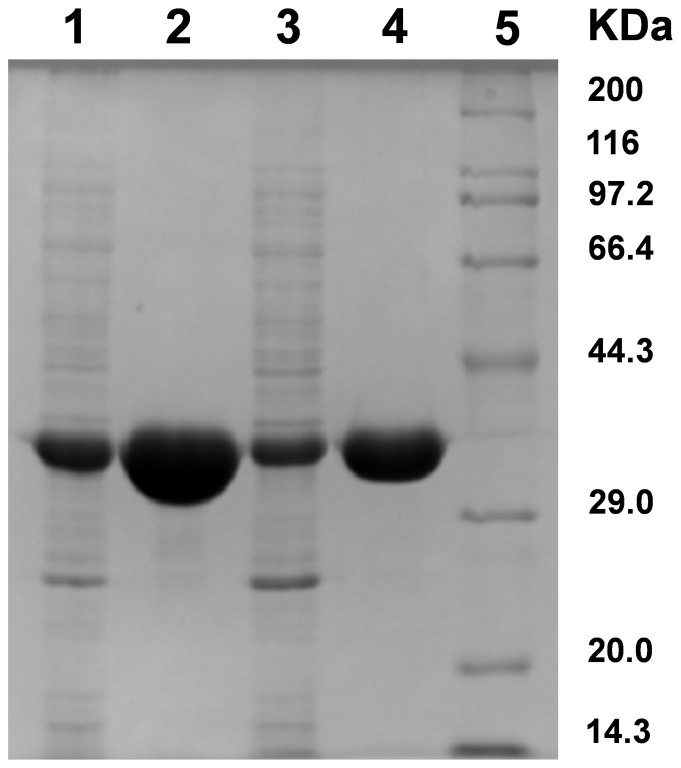
SDS-PAGE analysis of CgNal and EcNal. Lane 1, supernatant of pET28a-CgNal-Rosetta cell lysate, Lane2, purified CgNal, Lane 3, supernatant of pET28a-EcNal-Rosetta cell lysate, Lane 4, purified EcNal. CgNal and EcNal were cultivated, induced, purified and sampled under the same conditions.

CgNal gene of *C. glutamicum* ATCC 13032 (GenBank accession: NP_601846.1) encoding a 939 bp open reading frame, which corresponds to 312 amino acids, was cloned into pET-28a (+) vector with an N-terminal His6-tag (pET28a-CgNal). The *E. coli* strain harboring pET28a-CgNal exhibited the highest CgNal activity when OD_600_ reached 0.5 with 0.2 mM IPTG as inducer at 30°C (Fig. S1). Cultured under the optimal condition, CgNal was expressed mostly in soluble form. The purified CgNal showed a single band on SDS-PAGE image, corresponding to the molecular weight of CgNal (~33 KDa) (Fig. 2). When cultured and induced under the same conditions, CgNal showed higher expression level than EcNal as revealed by SDS-PAGE (Fig. 2). Up to 480 mg of purified CgNal could be obtained from 1 liter of *E. coli* Rosetta (DE3) pLysS culture, which is 2.23 fold higher than the highest expression level reported for LpNal (215 mg/L cell culture)^13^ and 4.33 fold higher than EcNal which was cultivated and purified under the same conditions. This advantage of CgNal over EcNal would make CgNal industrially more competitive than EcNal, which is commonly used in the industry.

### Biochemical characterization of recombinant CgNal

**Figure 3:**
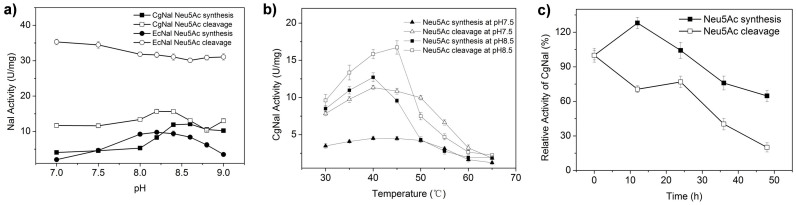
Effects of pH and temperature on CgNal activity. (a) Effects of pH on CgNal and EcNal, (b) Effects of temperature on CgNal at pH7.5 and pH8.5, (c) CgNal stability at pH 8.5, 40°C.

CgNal catalyzes a reversible aldo-cleavage/synthesis reaction. The effects of pH on CgNal were determined considering both Neu5Ac cleavage and synthesis activities. Interestingly, CgNal prefers more alkaline conditions (Fig. 3a) than most other NALs reported. In the Neu5Ac cleavage direction, the optimum pH of the CgNal was around pH 8.2 to 8.4, while it reached optimal Neu5Ac synthesis activity at pH 8.6. These values are obviously higher than the values described for other NALs, such as those from *E. coli*^28^, *P. multocida*^29^, *C. perfringens*^27^, *S. carnosus* TM300^14^ and *L. plantarum* WCF51^13^. None of those NALs obtain the optimum pH above pH 8.0 in either Neu5Ac synthesis or cleavage direction (Table 1).

In contrast to other NALs, CgNal is much more pH sensitive. For both Neu5Ac synthesis and cleavage reactions, CgNal showed sudden enhancement in its activities when the pH increased from 8 to 8.5. And the Neu5Ac synthesis activity was even higher than Neu5Ac cleavage activity when pH reached pH 8.8. This is the first time to report a NAL that favors aldo-condensation rather than cleavage. Considering this sharp change of CgNal's activity above pH 8, many CgNal characters were assayed at both pH 7.5 and pH 8.5.

The optimum temperature of CgNal was measured over a broad temperature range (30°C–65°C) at pH 7.5 and pH 8.5 for both Neu5Ac synthesis and cleavage directions (Fig. 3b). At pH 7.5 both directions adopted 40°C as the optimal reaction temperature, while at pH 8.5 the optimal temperature was 40°C and 45°C for Neu5Ac synthesis and cleavage direction, respectively. At both pHs, CgNal's activities dramatically decreased on both directions when temperature was above 50°C. The optimal temperature of CgNal is much lower than 65°C of EcNal^28^, 65–70°C of *C. perfringens* NAL^27^, 60–70°C of *S. carnosus* TM300 NAL^14^ and 70°C of *L. plantarum* WCFS1 NAL^13^ (Table 1), indicating that CgNal is not as thermo stable as other NALs.

The above results are in agreement with the following thermo stability studies (Fig. 3c). When CgNal was incubated at 40°C, pH 8.5 for 48 h, in Neu5Ac synthesis direction, CgNal kept 100% activity for the first 24 h but only remained 60% of its activity in 48h. In the meanwhile, Neu5Ac cleavage activity decreased even faster than Neu5Ac synthesis activity. Only 75% and 20% of cleavage activity remained in 24 h and 48 h, respectively. Interestingly, although CgNal is less thermo stable than other NALs, for the reversible aldo-cleavage/synthesis reaction, Neu5Ac synthesis would be the main reaction for CgNal in long time catalysis.

**Figure 4:**
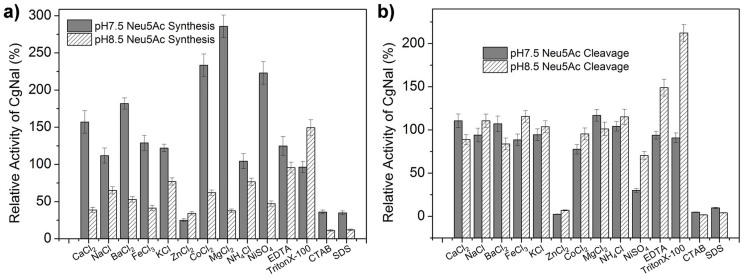
Effects of salts and detergents on CgNal activity. (a) Effects of salts and detergents to CgNal activity on Neu5Ac synthesis at pH7.5 and pH8.5 (b) Effects of salts and detergents to CgNal activity on Neu5Ac cleavage at pH7.5 and pH8.5.

The effects of metal ions or detergents on the enzymatic activities were tested on both Neu5Ac synthesis and cleavage at pH 7.5 and pH 8.5. We used the CgNal's Neu5Ac synthesis/cleavage activity at each pH without any metal ions' or detergents' addition as the standard to characterize the metal ions' or detergents' effects, respectively. For CgNal Neu5Ac synthesis activity, metal ions showed opposite effects at pH 7.5 and pH 8.5 (Fig. 4a). All metal ions, except Zn^2+^, activated the Neu5Ac synthesis at pH 7.5, while all metal ions inhibited the Neu5Ac synthesis at pH 8.5. There was no trend for the detergents' effects. EDTA showed activation activity at pH 7.5. TritonX-100 activated the Neu5Ac synthesis by 49.35% at pH 8.5. CTAB and SDS inhibited the synthesis activity on both pH. For Neu5Ac cleavage, metal ions can barely affect CgNal's enzymatic activity (Fig. 4b). The only exceptions were Zn^2+^ and Ni^2+^, with addition of 5 mM ZnCl_2_, CgNal remained only 2.41% and 6.95% cleavage activities at pH 7.5 and pH 8.5, while adding 5 mM NiSO_4_ to the reaction mixture, CgNal cleavage activities reduced to 30.07% and 70.34% at pH 7.5 and pH 8.5, respectively. EDTA and TritonX-100 hardly affected CgNal cleavage at pH 7.5 while activated the reaction at pH 8.5. CTAB and SDS showed inhibition at both pH.

### Kinetic parameters of CgNal

The kinetic parameters of CgNal were characterized in both Neu5Ac synthesis and cleavage directions at pH 7.5 and pH 8.5 (Table 1). For Neu5Ac synthesis activity, both ManNAc and pyruvate were tested as substrates. Consistent with the results above, kinetic parameters of CgNal were significantly affected by pH. At pH 7.5, CgNal did not show significant differences in *k_cat_* values, but the K_m_ values favored Neu5Ac synthesis. The CgNal K_m_ for Neu5Ac was found to be 33.5 mM, which is an order of magnitude higher than NALs from other organisms, meanwhile, the K_m_ for ManNAc and pyruvate was 53.3 mM and 14.7 mM, which is only 1/3~1/4 and half of the values from other organisms, respectively. This is the first time that the K_m_ values for Neu5Ac, ManNAc and pyruvate are in the same magnitude. This trend is more pronounced at pH 8.5. The K_m_ values for Neu5Ac, ManNAc and pyruvate were 87.7 mM, 92.1 mM and 72.4 mM, respectively. More interestingly, at pH 8.5 CgNal showed much higher activities for both Neu5Ac cleavage and synthesis. The *k_cat_* values for Neu5Ac, ManNAc and pyruvate were 44.2 S^−1^, 40.7 S^−1^ and 42.6 S^−1^, respectively. The *k_cat_* value for Neu5Ac cleavage was 4.4 fold of that from *Lactobacillus plantarum*^29^, which is the highest *k_cat_* value reported. And the *k_cat_* value for Neu5Ac synthesis was an order of magnitude higher than that from all other organisms. As a result, the *k_cat_*/K_m_ values of CgNal for ManNAc and pyruvate are 0.44 s^−1^mM^−1^ and 0.59 s^−1^mM^−1^, respectively, while *k_cat_*/K_m_ value for Neu5Ac is 0.50 s^−1^mM^−1^, on Neu5Ac cleavage. Compared to all previously reported NALs, CgNal exhibited the lowest *k*_cat_/K_m_ value for Neu5Ac and highest *k*_cat_/K_m_ value for ManNAc and pyruvate, which makes CgNal favor Neu5Ac synthesis the most.

### Sequence, structural and phylogenetic analysis

**Figure 5:**
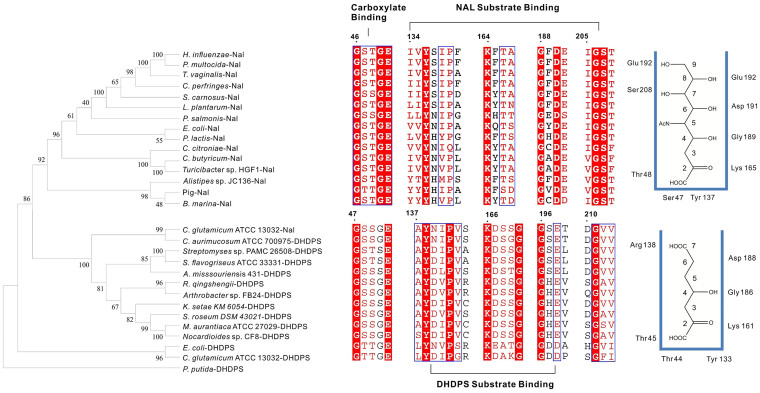
Phylogenetic tree of CgNal and alignment of key residues. The phylogenetic tree was constructed and visualized with MEGA 5.2^39^. BLAST identified DHDPS and NAL homologous to CgNal to construct the phylogenetic tree, *Pseudomonas putida* KDGDH (GenBank accession: P42233.1) was defined as the outgroup. The details of alignment outputs are shown in the middle of the figure. The schematic diagram showing modulation of residues within the active site of NAL and DHDPS is shown on the right side of the figure.

In spite of its high NAL activity, the protein sequence of CgNal was annotated in GenBank (accession: NP_601846.1) as a putative DHDPS. Actually, CgNal showed low sequence identities with either EcNal (23.7%) (PDB ID: 1NAL, 1FDY, 1FDZ and 1HL2)^15^,^16^,^17^ or EcDHDPS (26.4%). It showed only 22.8% identity with *Haemophilus influenzae* NAL (PDB ID: 1F5Z)^18^, 26.4% identity with E. coli DHDPS (PDB ID: 1DHP)^30^ and 22.9% identity with C. *glutamicum* ATCC 13032 DHDPS (PDB ID: 3CPR)^31^. Sequence alignment showed that CgNal contained conserved catalytic sites (Tyr138, Lys166) and the GXXGE motif (Gly47, Gly50 and Glu51) of NAL family (Table 2, Fig. S2). However, significant differences occurred at specific residues for NAL's substrate recognition. Asp191, Glu192, Ser208 of EcNal are responsible for Neu5Ac recognition and these three amino acids are conserved across all other NAL in the alignment (Fig. S2). Different residues were present at Neu5Ac recognition site of CgNal (Glu198, Thr199 and Val212). In the meanwhile, for DHDPS' substrate recognition, CgNal (Ser143, Gly196 and Glu198) showed higher similarity with EcDHDPS (Arg138, Gly186 and Asp188) (Fig. 5, Table 2).

Although, the identity of primary structure between CgNal and other NALs was fairly low, the predicted CgNal secondary structure showed much higher similarity with that of EcNal (Fig. S2). CgNal shared the common (β/α)_8_-barrel and the C-terminal extension of several α-helices, which are usual in the NAL family, includingNAL, DHDPS, KDGDH, HBPHA and other related enzymes^18^.

Obtaining 26.4% identity with EcDHDPS and 23.7% identity with EcNal, the phylogenetic analysis of CgNal would be very interesting. The phylogenetic tree of NAL and DHDPS was constructed by Mega 5.2 with NAL superfamily member KDGDH^18^ from *Pseudomonas putida* (GenBank accession: P42233.1) defined as the outgroup (Fig. 5). Not surprisingly, the phylogenetic tree could be divided into two groups, group1 for NAL and group2 for DHDPS (Fig. 5). But what do surprise us was that unlike previously reported NAL from *Clostridium perfringens*^27^, *Escherichia coli*^28^, Pig^11^, *Pasteurella multocida*^29^, *Staphylococcus carnosus* TM300^14^, *Lactobacillus plantarum* WCFS1^13^, *Trichomonas vaginalis*^32^, *Haemophilus influenzae*^33^, CgNal had higher similarity with DHDPS and it was classfied into the DHDPS group. Sequence alignments of group1 and group2 were shown in Fig. S3 and the DHDPS group showed more conserved residues than the NAL group.

### Substrate specificity of CgNal

**Figure 6:**
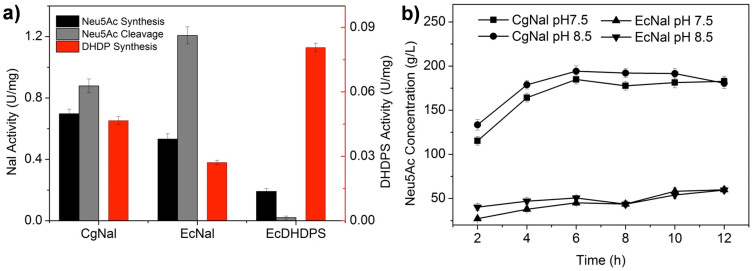
(a) Comparison of enzyme activity with different substrate of CgNal, EcNal and EcDHDPS. (b) Synthesis of Neu5Ac by CgNal and EcNal. In a reaction mixture containing 0.8 M ManNAc, 2 M pyruvate and 0.5 M Tris-HCl (pH 7.5 and pH 8.5) purified EcNal or CgNal was added to initiate the synthesis.

Although CgNal showed very high NAL acitivity, it is more belonging to DHDPS according to sequence, structural and phylogenetic analysis. So we compared the enzyme activities of CgNal, EcNal and EcDHDPS with both substrates of NAL and DHDPS (Fig. 6a). Neu5Ac and ManNAc were used for characterizing NAL's activities in Neu5Ac synthesis and cleavage directions, respectively. ASA was used for characterizing their DHDPS activities. CgNal, EcNal and EcDHDPS were active to both DHDPS' and NAL's substrates. Using ASA as substrate, CgNal showed only half the activity of EcDHDPS but twice the activity of EcNal. For NAL activity analysis, in Neu5Ac synthesis direction, CgNal showed higher activity than EcNal, while in the Neu5Ac cleavage direction, the activity of CgNal was much lower than EcNal. This special property made CgNal a potential enzyme for industrial Neu5Ac synthesis.

## Discussion

Enzymes from aldolase Class-I superfamily share a common structural framework of (α/β)_8_ barrel, but catalyze different reactions on separate biochemical pathways^18^. Among all the members of this superfamily, NAL and DHDPS are best-characterized. NAL takes in charge of the regulation of intracellular sialic acid by catalyzing the cleavage of Neu5Ac to produce ManNAc and pyruvate, while DHDPS catalyzes the aldol condensation of pyruvate and ASA, which is the first step in the biosynthesis of lysine via the diaminopimelate pathway. In this paper, we describe the cloning, expression and biochemical characterization of a novel NAL (CgNal) from the “GRAS” organism *C. glutamicum* ATCC 13032, which shows bi-function as NAL and DHDPS.

According to the primary sequence alignment, CgNal obtains 26.4% identity with EcDHDPS and 23.7% identity with EcNal. The phylogenetic analysis shows that CgNal is evolutionarily more related to DHDPS (Fig. 5). But the enzyme activity assay with different substrates of NAL (Neu5Ac synthesis/cleavage) and DHDPS illustrates that CgNal obtains the higher enzymatic activity as NAL in both cleavage and synthesis directions than that as DHDPS. More importantly, compare to all previously reported NALs, CgNal has higher conversion speed and conversion rate towards Neu5Ac synthesis, showing great potential in biotechnological application for industrial synthesis of sialic acid.

Neu5Ac is an important 9-carbon amino sugar with huge market demand in pharmaceutical and food industry and NAL catalyzes the rate-limiting step for Neu5Ac synthesis^34^. NALs catalyze the reversible aldol cleavage of Neu5Ac to form pyruvate and ManNAc. For the reversible reaction, all the NALs reported previously favored the Neu5Ac cleavage, resulting in the low yield for industrial Neu5Ac synthesis. CgNal illustrates the highest conversion speed and conversion rate towards Neu5Ac synthesis. At pH 7.5, the *k_cat_* values of CgNal for Neu5Ac and ManNAc are similar to other NALs, but *k_cat_* for pyruvate is about 3 times to others. At pH 8.5, the turnover rate increases dramatically for all substrates. The *k_cat_* values for Neu5Ac, ManNAc and pyruvate are 44.2 s^−1^, 40.7 s^−1^ and 42.6 s^−1^, which are 3~20 times to those of other NALs. CgNal exhibits low affinity to Neu5Ac, which is the substrate for cleavage reaction. K_m_ values of CgNal are 33.5 mM and 87.7 mM at pH 7.5 and pH 8.5, respectively. It is an order of magnitude higher than the K_m_ values of NALs, which are 3.5 mM, 4.9 mM, 3.2 mM, 2 mM and 1.8 mM from *E. coli*, *P multocida*, *C. perfringens*, *S. carnosus* TM300 and *L. plantarum* WCFS1 respectively (Table 1). Compared to other NALs, CgNal's affinities to ManNAc and pyruvate, the substrates for Neu5Ac synthesis, are relatively high. The K_m_ values of CgNal are 53.3 mM and 92.1 mM to ManNAc at pH 7.5 and pH 8.5, respectively, which are only 1/4 to half of the K_m_ values of NALs to ManNAc from other organisms. Therefore, at pH 8.5 CgNal shows the best enzymatic efficiency on Neu5Ac synthesis. The *k_cat_*/K_m_ values of CgNal for ManNAc and pyruvate are 0.44 s^−1^mM^−1^ and 0.59 s^−1^mM^−1^, respectively, on Neu5Ac synthesis, while *k_cat_*/K_m_ value for Neu5Ac is 0.50 s^−1^mM^−1^, on Neu5Ac cleavage. Compared to the previously reported NALs, whose *k_cat_*/K_m_ value for Neu5Ac cleavage is one or two orders of magnitude to those for Neu5Ac synthesis, CgNal favors Neu5Ac synthesis the most (Table 1). Therefore, CgNal shows extraordinary catalysis properties for Neu5Ac synthesis. 194 g/L Neu5Ac (0.63 M) was obtained from 2 M pyruvate and 0.8 M ManNAc with purified CgNal as catalyst (Fig. 6b), which was the highest among all reported Neu5Ac yield of 12.3 g/L^7^, 19.1 g/L^25^, 18.32 g/L^35^, 59 g/L^36^, 61.3 g/L^34^, 122.3 g/L^37^.

Although CgNal illustrates the best properties for Neu5Ac synthesis, the phylogenetic analysis shows that CgNal is evolutionarily more related to DHDPS. As shown in Fig. 5, the phylogenetic tree of CgNal and other putative NAL and DHDPS, were clearly divided into two groups: the NAL group and DHDPS group. CgNal was grouped into the DHDPS group (Fig. 5 and Fig. S3). The alignment of the key residues from each group revealed different patterns in various motifs^13^. The catalytic site in CgNal (Tyr 138 and Lys 166, residues in CgNal responsible for aldo-cleavage) showed homogeneity with both NAL and DHDPS. Slight difference was observed for “GXXGE” carboxylate binding motif. The α-keto acid binding motif is highly conserved in all the NALs and the DHDPSs subfamily of (β/α)_8_ enzymes. But most enzymes of NAL subfamily adopt “GSTGE” motif in NAL group, while CgNal adopts the “GSSGE” motif shared by most of the DHDPS. In contrast, the sugar binding motif of the NAL group and the DHDPS group showed most distinguished differences due to different substrate specificity of these two groups. In NAL group, the Neu5Ac binding sites are relatively conservative. For example, in EcNal, Asp191, Ser208 and Glu192 interact with the hydroxyl groups O_6–9_. But CgNal equivalents Glu198, Thr199 and Val212 showed higher homogeneity with DHDPS group (Asp188, Ala189 and Val205 of EcDHDPS) (Fig. 5 and Table 2). In EcDHDPS, Asp188 is responsible for interacting with the ammonium group of ASA, while Ala189 and Val205, the equivalents of EcNal residues Glu192 and Ser208, showed no specific interaction with the pyruvate-ASA complex^18^. Arg138 in EcDHDPS was assigned to interact with the carboxylate group at the C_7_ position of the bound complex^18^. Absent in CgNal, Ser143 replaced Arg instead, while a conserved leucine shows in NAL group, which exhibits no interactions with the Neu5Ac (Fig. 5 and Table 2). This phenomenon elucidates that CgNal is phylogenetically more related to DHDPS, but during the evolution, CgNal might adopt a unique set of residues for either Neu5Ac or ASA recognition.

Although there is no three-dimensional structure for either CgNal or CgNal-substrate complex available to confirm our hypothesis, the comparison among enzyme activities with different substrates of NAL (Neu5Ac synthesis/cleavage) and DHDPS supports our assumption. CgNal activities towards the substrates of both NAL (ManNAc and Neu5Ac) and DHDPS (ASA) were assayed with EcNal and EcDHDPS as controls. CgNal, EcNal and EcDHDPS all showed detectable activities towards substrates of NAL and DHDPS (Fig. 6a), possibly because they belong to the same superfamily (with the same catalysis residues). But these aldolases showed different preferences towards the substrates. EcDHDPS demonstrated the highest DHDPS activity and EcNal showed the highest Neu5Ac cleavage activity. With the higher similarity to DHDPS, CgNal performed higher DHDPS activity than EcNal. But still CgNal illustrated high Neu5Ac cleavage activity and the highest Neu5Ac synthesis activity among all three. This result, combined with the analysis of the CgNal residues that are responsible for recognition of NAL's and DHDPS' substrate, indicated that CgNal might adopt a unique set of residues for substrates recognition, favoring Neu5Ac synthesis.

As a unique enzyme showing bi-function as NAL and DHDPS, CgNal illustrated a distinctive set of key residues for substrates recognition. Moreover, the outstanding Neu5Ac synthesis activity of CgNal made it an interesting enzyme for enzymatic research and a promising catalyst in industrial synthesis of Neu5Ac. Due to the limited information we can extract from sequence alignment, further elucidation of the catalysis mechanism of CgNal can be achieved by resolving the crystal structure of CgNal itself and CgNal-substrate complex in our future study.

## Methods

### Strains, plasmids and chemicals

*C. glutamicum* ATCC 13032 strain was obtained from ATCC (Manassas, VA). *E.* *coli* DH5α and *E.* *coli* Rosetta (DE3) pLysS strains were from Promega (Shanghai, China). pET-28a(+) vector were from Novagen (Darmstadt, Germany). Restriction enzymes, DNA polymerase, genomic DNA extraction kit, DNA markers, protein markers were from Takara (Dalian, China). CloneExpress one-step cloning kit was from Vazyme (Shanghai, China). *N*-acetyl-D-mannosamine and *N*-acetylneuraminic acid were from Sigma-Aldrich (Shanghai, China). All other chemicals were of analytical grade and commercially available.

### Cloning of CgNal gene, EcDHDPS gene and EcDHDPR gene

Genomic DNA of *C. glutamicum* ATCC 13032 and *E. coli* Rosetta (DE3) pLysS were isolated as PCR template, respectively, using Takara genomic DNA extraction kit. The CgNal gene sequence (GenBank accession: NP_601846.1), which was deposited in NCBI database as a putative DHDPS, was amplified from *C. glutamicum* genomic DNA with primers specific for its 5′ and 3′ ends. The *E. coli* *dapA* gene, encoding a DHDPS, and *dapB* gene, encoding a dihydrodipicolinate reductase (DHDPR) protein, were amplified from *E. coli* genomic DNA with primers specific for each 5′ and 3′ ends, incorporating *Eco*RI and *Hin*dIII restriction sites, respectively. All primers (Table S1) were designed according to Vazyme CloneExpress handbook with homology arms on 5′ terminal. The PCR product was purified and proportionally mixed with pre-linearized pET-28a (+) vector to construct pET28a-CgNal pET28a-EcDHDPS and pET28a-EcDHDPR following CloneExpress protocol. The constructed plasmids were sequenced, amplified and transformed into *E. coli* Rosetta (DE3) pLysS cells to obtain recombination strains pET28a-CgNal-Rosetta, pET28a-EcDHDPS-Rosetta and pET28a-EcDHDPR-Rosetta.

### Expression and purification of CgNal, EcNal, EcDHDPS and EcDHDPR

The recombinant strain pET28a-EcNal-Rosetta harboring Nal cloned from *E. coli* was constructed in our previous research^34^ and used as a control in this study. Recombinant strains pET28a-CgNal-Rosetta, pET28a-EcNal-Rosetta, pET28a-EcDHDPS-Rosetta and pET28a-EcDHDPR-Rosetta were cultured in 200 mL LB media (containing 10 μg·ml^−1^ kanamycin and 34 μg·ml^−1^ chloromycetin) overnight at 37°C, 220 rpm, and were induced at optical density (OD_600_) of 0.6–0.8, with 0.5 mM β-D-1-thiogalactopyranoside (IPTG) at 30°C overnight. Cells were harvested by centrifugation at 4000 g for 10 min at 4°C (Eppendorf Centrifuge 5810R, Germany). Prior to sonication, the cell pellets were washed twice with lysis buffer (100 mM Tris-HCl, pH 7.5). Resuspended with 10 ml of lysis buffer, cells were sonicated at 200 W with 3 s on and 5 s off for 60 times, using Ultrasonic Disruptor JY92-11 (NingBo Scientz, China). Clarified by centrifugation at 9000 g for 20 min at 4°C, the supernatants were loaded onto Ni–NTA Agarose column (Column purchased from Bio-Rad was 1 cm in diameter and packed with the total volume of 1 ml Ni–NTA Agarose beads purchased from Qiagen). After washing the column with 40 ml of washing buffer (100 mM Tris-HCl, 20 mM imidazole, pH 7.5), enzymes were eluted with 2 ml of eluting buffer (100 mM Tris-HCl, 500 mM imidazole, pH 7.5). Purified CgNal, EcNal, EcDHDPS and EcDHDPR were dialyzed in 100 mM Tris-HCl, pH 7.5, for 24 h, at 4°C prior to any assay. Purities of enzymes were estimated to be >90% by Coomassie Blue G-250 stained 12% sodium dodecyl sulfate polyacrylamide gel electrophoresis (SDS-PAGE).

### Optimization of CgNal production

Effects of induction conditions, including optical density (OD_600_) before induction, β-D-1-thiogalactopyranoside (IPTG) concentration and induction temperature on CgNal expression were examined. It was performed by changing OD_600_ between 0.1–1.5, IPTG concentrations between 0.1–0.8 mM and induction temperature between 20°C–40°C, respectively, while other conditions were fixed. Crude enzyme activities (Neu5Ac cleavage, pH 7.5) after centrifugation and sonication were assayed to determine the effects.

### Enzyme assay of CgNal and EcNal

Both enzyme activity of CgNal and EcNal was assayed by measuring its ability to condense ManNAc and pyruvate into Neu5Ac (Neu5Ac synthesis) as well as its ability to cleave Neu5Ac into ManNAc and pyruvate (Neu5Ac cleavage). The Neu5Ac synthesis reaction mixture consisted of 0.1 M pyruvate, 0.05 M ManNAc and 0.1 M Tris-HCl (pH 7.5 or 8.5) and the Neu5Ac cleavage reaction mixture contained 0.05 M Neu5Ac and 0.1 M Tris-HCl (pH 7.5 or 8.5). Protein concentration was determined by Bradford method^38^, CgNal or EcNal was added to 1 mL reaction mixture to final concentration of 130 μg/ml. After incubation at 37°C for 10 min, reactions were terminated by boiling the mixture for 5 min, centrifuged at 12000 × g for 10 min and filtrated through 0.22 μm membrane. The concentrations of the substrates and the products were analyzed by high performance liquid chromatography (HPLC). Samples were analyzed on an Agilent 1200 system equipped with a Bio-Rad Aminex HPX-87H column (300 × 7.8 mm) using a refractive index detector. The mobile phase consisted of 5 mM H_2_SO_4_ at 0.6 mL/min, 55°C. All tests were performed in triplicate and 1 unit of enzyme activity was defined as the amount of enzyme needed to produce 1 μmol product per min.

### Effects of pH and temperature on CgNal

The effect of pH on CgNal was determined using the following buffers: 0.1 M Tris-HCl (pH 7–pH 8.8) and 0.1 M glycine-NaOH (pH 9). Optimum pH for CgNal was determined by measuring CgNal activities in reaction systems with different pH. Optimum temperature of CgNal was determined by measuring CgNal activities (at pH 8.5) under different reaction temperatures (30°C–65°C). CgNal stability was determined by measuring residual Neu5Ac activities after incubating the enzyme at pH 8.5, 40°C for 48 h.

### Effects of salts and detergents on CgNal

Effects of salts and detergents on CgNal were determined by measuring Neu5Ac synthesis and cleavage activities of CgNal in 100 mM Tris-HCl buffer (at pH 7.5 and pH 8.5) in the presence of 5 mM salts and detergents, including CaCl_2_, NaCl, BaCl_2_, FeCl_3_, KCl, ZnCl_2_, CoCl_2_, MgCl_2_, NH_4_Cl, NiSO_4_, EDTA, TritonX-100, CTAB and SDS. Reaction mixture without salts and detergents was used as control.

### Kinetic parameters of CgNal and EcNal

Kinetic parameters (K_m_, V_max_) for CgNal and EcNal were assayed by measuring their activities at the presence of various concentrations of substrates. For Neu5Ac synthesis reaction, ManNAc concentration was fixed at 50 mM with pyruvate concentrations varied (20 mM–450 mM) to determine kinetic parameters for pyruvate; and pyruvate concentration was fixed at 100 mM with ManNAc concentration varied (20 mM–450 mM) to determine kinetic parameters for ManNAc. As for Neu5Ac cleavage reaction, Neu5Ac concentration was varied from 1 mM to 200 mM to determine kinetic parameters for Neu5Ac. K_m_ and V_max_ were calculated with GraphPad Prism 5.0 (GraphPad, San Diego).

### Sequence, structural and phylogenetic analysis

Basic Local Alignment Search Tool (BLAST) of NCBI was used to identify proteins homologous to CgNal, and MEGA 5.2^39^ was used to construct the phylogenetic tree with bootstrap value calculated after 1000 generations. CgNal subunit structure was modeled by Phyre2 server at intensive mode^40^. Sequences were aligned with ClustalW^41^ and ESPript^42^.

### Substrate specificity of CgNal

CgNal's activity towards the substrates of both NAL (ManNAc and Neu5Ac) and DHDPS (ASA) were assayed with EcNal and EcDHDPS as controls. The activities of CgNal with ManNAc and Neu5Ac as substrate were assayed as mentioned in Enzyme assay of CgNal section. The activity of CgNal with ASA as substrate was measured using a coupled enzymatic assay as described by Yugari and Gilvarg^43^. All enzymatic assays were replicated 3 times.

## Supplementary Material

Supplementary Information Characterization of a novel N-acetylneuraminic acid lyase favoring N-acetylneuraminic acid synthesis

## Figures and Tables

**Figure 1:**
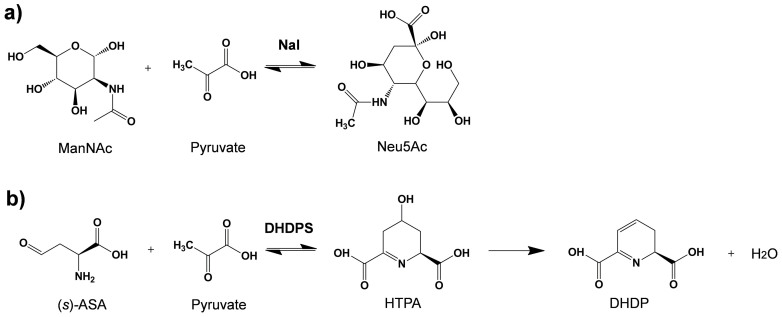
Reactions catalyzed by (a) Nal and (b) DHDPS.

**Table 1:** Enzyme characters and Kinetic parameters of NAL from different organisms

	Neu5Ac Cleavage						Neu5Ac Synthesis										
	Neu5Ac						ManNAc				Pyruvate						
	K_m_ (mM)	Vmax (U/mg)	*k*_cat_ (s^−1^)	*k*_cat_/K_m_ (s^−1^mM^−1^)	Optimum pH	Optimum Temperature(°C)	K_m_ (mM)	V_max_ (U/mg)	*k*_cat_ (s^−1^)	*k*_cat_/K_m_ (s^−1^mM^−1^)	K_m_ (mM)	V_max_ (U/mg)	*K*_cat _(s^−1^)	*k*_cat_/K_m_ (s^−1^mM^−1^)	Optimum pH	Optimum Temperature(°C)	References
***C. glutamicum***	33.50	16.74	9.30	0.28	8.4–8.8	40	53.30	10.20	5.67	0.11	14.70	10.98	6.10	0.41	8.2–8.4	40	This study
**ATCC**																	
**13032 (pH 7.5)**																	
***C. glutamicum***	87.70	79.60	44.20	0.50		45	92.10	73.20	40.67	0.44	72.40	76.64	42.58	0.59		40	This study
**ATCC**																	
**13032 (pH 8.5)**																	
***Escherichia coli***	3.50	151.10	83.11	23.75	-	-	287.1	35.28	19.4	0.07	206.1	40.42	22.23	0.11			This study
**(pH 7.5)**																	
***Escherichia coli***	3.60	154.50	84.97^a^	23.60	7.7	75	-	-	-	-	-	-	-	-	7.7	-	[28]
***Escherichia coli***	2.50	18^b^	10	4	-	-	180	16.36^b^	9	0.05	22	3.24^b^	1.80	0.08	-	-	[29]
***Pasteurella multocida***	4.90	29^b^	16	3.27	7.5–8.0	-	220	20^b^	11	0.05	23	3.44^b^	1.90	0.08	7.5–8.0	-	[29]
***Clostridium perfringens***	3.20	27.50	16.04^c^	5.01	7.6	65–70	-	-	-	-	-	-	-	-	-	-	[27]
***Staphylococcus carnosus***	2	6.85^d^	4	2	7	60–70	149	7.68^d^	4.50	0.03	14	4.93^d^	2.90	0.21	7	50	[14]
**TM300**																	
***Lactobacillus plantarum***	1.80	18.27^b^	10.08	5.60	7.5	70	160	8.72^b^	4.80	0.03	19.90	3.96^b^	2.19	0.11	7.50	60	[13]
**WCFS1**																	

^a^Calculated according to V_max_ and MW = 33 KDa;

^b^Calculated according to *k*_cat_ and MW = 33 KDa;

^c^Calculated according to V_max_ and MW = 35 KDa;

^d^Calculated according to *k*_cat_ and MW = 35 KD.

**Table 2:** Comparison of key residues of CgNal, EcNal and EcDHDPS

				NAL Sugar Recognition			
enzyme	catalysis		DHDPS Sugar Recognition				
CgNal	Tyr138	Lys166	Ser143	Gly196	Glu198	Thr199	Val212
EcNal	Tyr137	Lys165	(Leu142)	Gly189	Asp191	Glu192	Ser208
EcDHDPS	Tyr133	Lys161	Arg138	Gly186	Asp188	(Ala189)	(Val205)
