# Corrigendum: Characterization of a novel *N*-acetylneuraminic acid lyase favoring industrial *N*-acetylneuraminic acid synthesis process

**DOI:** 10.1038/srep42268

**Published:** 2017-04-28

**Authors:** Wenyan Ji, Wujin Sun, Jinmei Feng, Tianshun Song, Dalu Zhang, Pingkai Ouyang, Zhen Gu, Jingjing Xie

Scientific Reports 5: Article number: 0934110.1038/srep09341; published online: 03 23 2015; updated: 04 28 2017

The original version of this article contained an error in testing the kinetic parameters of CgNal towards pyruvate, in which the concentration of ManNAc (50 mM) was not in excess. This error was corrected by re-running the assay in the presence of excessive ManNAc (180 mM). The corrected kinetic parameters of CgNal towards pyruvate were updated in Table 1 of the manuscript. These changes do not change the conclusions of the article.

Apparent kinetic parameters (Km, Vmax) of CgNal towards pyruvate were re-assayed as follows. ManNAc concentration was fixed at 180 mM with pyruvate concentrations varied (20 mM–450 mM) to determine kinetic parameters for pyruvate. The reaction was run at 37 °C, at pH 7.5 and pH 8.5 and terminated by heat inactivation at 95 °C for 5 min. After centrifugation at 10000 rpm for 10 min at 4 °C, the supernatant was filtered using a 0.22 μm filter. Concentration of Neu5Ac was quantified by HPLC using a Bio-Rad Aminex HPX-87H (300 × 7.8 mm) column with 5 mM sulfuric acid solution as mobile phase. Kinetic parameters were calculated with GraphPad Prism 5.0.

The original and revised kinetic parameters appear below as Table 1 and Table 2 respectively.

In addition, the expression “favoring *N*-acetylneuraminic acid synthesis”, which was based on the enzymatic reaction under real industrial process conditions (non-equilibrium and non-standard conditions), caused some confusion about “shifting chemical equilibrium”. We have clarified the title and related expressions throughout the manuscript.

1. The title “Characterization of a novel *N*-acetylneuraminic acid lyase favoring *N*-acetylneuraminic acid synthesis” now reads “Characterization of a novel *N*-acetylneuraminic acid lyase favoring industrial *N*-acetylneuraminic acid synthesis process”.

2. Enzyme kinetic parameters “K_m_”, “V_max_”, “*k*_*cat*_” and “*k*_*cat*_*/K*_*m*_” now read “apparent K_m_”, “apparent V_max_”, “apparent *k*_*cat*_” and “apparent *k*_*cat*_*/K*_*m*_” in the main text.

3. In the abstract, “Due to the equilibrium favoring Neu5Ac cleavage……” now reads “Due to the high Neu5Ac cleavage activity in most isozyme forms……” and “……, which makes CgNal favor Neu5Ac synthesis the most” now reads “……, which makes CgNal favor industrial Neu5Ac synthesis process in a non-equilibrium condition”.

4. In the introduction section: “yield” is replaced with “space-time yield” to emphasize the non-equilibrium nature of industrial reactions; “……of favoring Neu5Ac synthesis in the reversible reaction” now reads “……of favoring industrial Neu5Ac synthesis in the reversible reaction under an optimized and non-equilibrium condition”.

5. In the “Biochemical characterization of recombinant CgNal” section: “……a NAL that favors aldo-condensation rather than cleavage” now reads “……a NAL that favors aldo-condensation rather than cleavage under an optimized condition”.

6. In the “Kinetic parameters of CgNal” section: “The kinetic parameters of CgNal were characterized in both Neu5Ac synthesis and cleavage directions at pH 7.5 and pH 8.5……” now reads “To evaluate the kinetic properties of CgNal, the second-order reaction was simplified as a pseudo first-order reaction by adding excessive amount of one substrate while varying the concentration of the other. To differentiate the values from theoretical kinetic parameters, we named them as apparent kinetic parameters to avoid confusion. The apparent kinetic parameters of CgNal were characterized in both Neu5Ac synthesis and cleavage directions at pH 7.5 and pH 8.5……”; “……which makes CgNal favor Neu5Ac synthesis the most”. now reads “……which makes CgNal favor industrial Neu5Ac synthesis process the most. It should be noted that unlike single-substrate reversible reactions, most multi-substrate reversible reactions could never attain equilibrium^15^. The shift in the apparent kinetic parameters does not imply a change in chemical equilibrium that is only associated with the free energy difference between the substrates and products”.

7. In the discussion section: “CgNal illustrates the highest conversion speed and conversion rate towards Neu5Ac synthesis”. now reads “……CgNal illustrates the highest conversion speed and conversion rate towards Neu5Ac synthesis under an optimized condition”.; “yield” is replaced with “space-time yield” to emphasized that non-equilibrium nature of industrial reactions; “……CgNal favors Neu5Ac synthesis the most. Therefore, CgNal shows extraordinary catalysis properties for Neu5Ac synthesis”. now reads “……CgNal favors industrial Neu5Ac synthesis process the most”.; “Although CgNal illustrates the best properties for Neu5Ac synthesis” now reads “Although CgNal illustrates the best properties for industrial Neu5Ac synthesis”; “favoring Neu5Ac synthesis” now reads “……favoring industrial Neu5Ac synthesis process”.

These errors have now been corrected in the HTML version of this Article.

## Figures and Tables

**Table 1:** Original kinetic parameters.

	Neu5Ac Synthesis			
	Pyruvate			
	K_m_ (mM)	V_max_ (U/mg)	*k*_cat_ (s^−1^)	*k*_cat_/K_m_ (s^−1^mM^−1^)
*C. glutamicum* ATCC 13032 (pH 7.5)	14.70	10.98	6.10	0.41
*C. glutamicum* ATCC 13032 (pH 8.5)	72.40	76.64	42.58	0.59

**Table 2:** Revised kinetic parameters.

	Neu5Ac Synthesis			
	Pyruvate			
	K_m_ (mM)	V_max_ (U/mg)	*k*_cat_ (s^−1^)	*k*_cat_/K_m_ (s^−1^mM^−1^)
*C. glutamicum* ATCC 13032 (pH 7.5)	18.85	16.30	9.06	0.48
*C. glutamicum* ATCC 13032 (pH 8.5)	31.28	19.43	10.80	0.34

