# Supplementary material for: Characterization of a novel N-acetylneuraminic acid lyase favoring N-acetylneuraminic acid synthesis
Source: Sci Rep. 2015 Mar 23;5:9341. doi: 10.1038/srep09341 (PMC5380162; doi:10.1038/srep09341)
Supplement: Supplementary Information — Characterization of a novel N-acetylneuraminic acid lyase favoring N-acetylneuraminic acid synthesis [file srep09341-s1.doc]

***Supporting Information***

**Characterization of a novel *N*-acetylneuraminic acid lyase favoring *N*-acetylneuraminic acid synthesis**

Wenyan Ji ^1,2,3,*^, Wujin Sun^4,*^, Jinmei Feng^5^, Tianshun Song^2^, Dalu Zhang^6^ , Pingkai Ouyang ^1,2,3^, Zhen Gu^4^, Jingjing Xie^1,2,3^

^1^State Key Laboratory of Materials-Oriented Chemical Engineering, Nanjing, PR China, ^2^College of Life Science and Pharmaceutical Engineering, Nanjing Tech University, Nanjing, PR China, ^3^National Engineering Technique Research Center for Biotechnology, Nanjing, PR China, ^4^Joint Department of Biomedical Engineering, University of North Carolina at Chapel Hill and North Carolina State University, Raleigh, United States, ^5^Department of Pathogenic Biology, School of Medicine, Jianghan University, Wuhan, China, ^6^International Cooperation Division, China National Center for Biotechnology Development, Beijing, PR China

Correspondence and requests for materials should be addressed to J.X. (xiej@njtech.edu.cn)

^*^These authors contributed equally to this work.

Fig. S1. Optimization of CgNal expression conditions. a) Effects of IPTG concentration on CgNal activity. b) Effects of induction temperature on CgNal activity. c) Effects of optical density (OD_600_) before induction on CgNal activity. Reactions were performed in triplicate, and error bars represent the standard error of the mean.


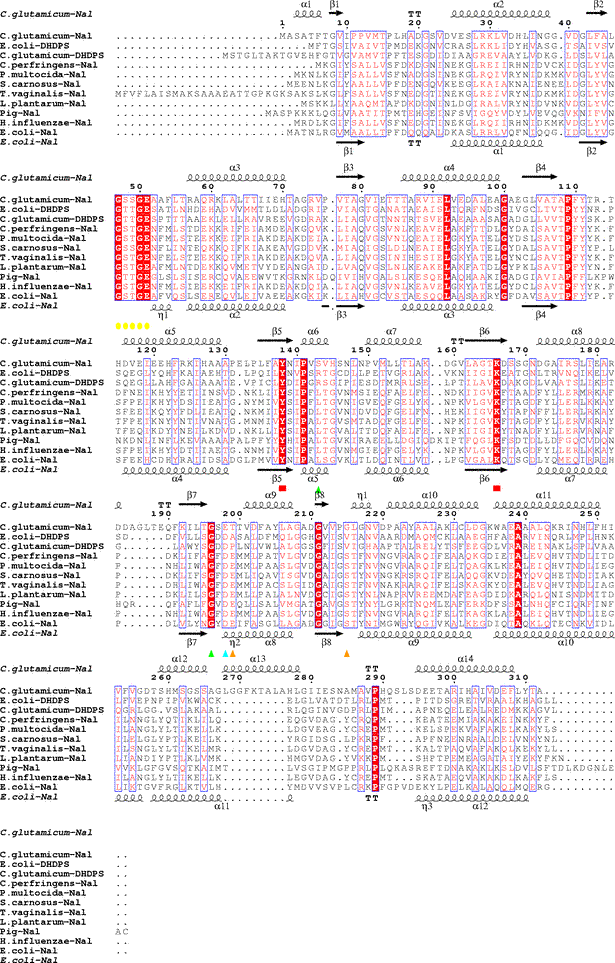


Fig. S2. Sequence alignment of Nal. Secondary structures were annotated according to 1NAL and CgNal structure simulated by Phyre2^1^. The catalytic Lys165 and Tyr137 (*E. coli* numbering) were marked with red squares, the GXXGE motif were marked with yellow circles. Residues for Nal substrate recognition (Glu192, Ser208 in EcNal numbering) were marked with orange triangles, residues for DHDPS substrate recognition (Arg 138, Gly186 in E. coli DHDPS numbering) were marked with green triangles and the common Asp 191 shared by EcNal and E. coli DHDPS substrate recognition was marked with cyan triangle. Sequences used in this alignment were *E. coli* DHDPS (PDB ID: 1DHP)^2^, C. *glutamicum* ATCC 13032 DHDPS (PDB ID: 3CPR) ^3^, *Clostridium perfringens* Nal(GenBank accession: CAA73375)^4^, *Pasteurella multocida* Nal (GenBank accession: YP_006240912)^5^, *Staphylococcus carnosus* TM300 Nal (GenBank accession: YP_002635477)^6^, *Trichomonas vaginalis* Nal (GenBank accession: AAB42182) ^7^, *Lactobacillus plantarum* WCFS1 Nal (GenBank accession: YP_004891044)^8^, Pig Nal (GenBank accession: NP_999236.1) ^9^, *Haemophilus influenzae* Nal (PDB ID: 1F5Z)^10^, EcNal (PDB ID: 1NAL,1FDY, 1FDZ and 1HL2) ^11-13^.

Fig. S3. Alignment of sequences in a) Nal and b) DHDPS groups.

Table S1. Primers design of CgNal, EcDHDPS and EcDHDPR

|  | **Primers** | **restriction sites** |
| --- | --- | --- |
| **CgNal** | S: GACAGCAAATGGGTCGCGGATCCATGGCTTCCGCAACTTTCACCG | *Bam*HI/*Hin*dIII |
|  | A:TGCTCGAGTGCGGCCGCAAGCTTTTAAGCGGTGTACAGGAATTCATC |  |
| **EcDHDPS** | S: GCAAATGGGTCGCGGATCCGAATTCATGTTCACGGGAAGTATT | *Eco*RI/*Hin*dIII |
|  | A: CTCGAGTGCGGCCGCAAGCTTTTACAGCAAACCGG |  |
| **EcDHDPR** | S: GCAAATGGGTCGCGGATCCGAATTCatgcatgatgcaaacatcc | *Eco*RI/*Hin*dIII |
|  | A: GGTGCTCGAGTGCGGCCGCAAGCTTTTACAAACTATTGAGATCA |  |

1. Kelley, L.A. & Sternberg, M.J.E. Protein structure prediction on the Web: a case study using the Phyre server. *Nat. Protoc.* **4**, 363-371 (2009).

2. Devenish, S.R.A., Gerrard, J.A., Jameson, G.B. & Dobson, R.C.J. The high-resolution structure of dihydrodipicolinate synthase from Escherichia coli bound to its first substrate, pyruvate. *Acta Crystallogr. Sect. F* **64**, 1092-1095 (2008).

3. Rice, E.A., Bannon, G.A., Glenn, K.C., Jeong, S.S., Sturman, E.J. & Rydel, T.J. Characterization and crystal structure of lysine insensitive Corynebacterium glutamicum dihydrodipicolinate synthase (cDHDPS) protein. *Arch. Biochem. Biophys.* **480**, 111-121 (2008).

4. Krüger, D., Schauer, R. & Traving, C. Characterization and mutagenesis of the recombinant N-acetylneuraminate lyase from Clostridium perfringens. *Eur. J. Biochem.* **268**, 3831-3839 (2001).

5. Li, Y., Yu, H., Cao, H., Lau, K., Muthana, S., Tiwari, V.K., Son, B. & Chen, X. Pasteurella multocida sialic acid aldolase: A promising biocatalyst. *Appl. Microbiol. Biotechnol.* **79**, 963-970 (2008).

6. García García, M.I., Sola Carvajal, A., García Carmona, F. & Sánchez Ferrer, Á. Characterization of a Novel N-Acetylneuraminate Lyase from Staphylococcus carnosus TM300 and Its Application to N-Acetylneuraminic Acid Production. *J. Agr. Food Chem.* **60**, 7450-7456 (2012).

7. Meysick, K.C., Dimock, K. & Garber, G.E. Molecular characterization and expression of a N-acetylneuraminate lyase gene from Trichomonas vaginalis. *Mol. Biochem. Parasitol.* **76**, 289-92 (1996).

8. Sanchez-Carron, G., Garcia-Garcia, M.I., Lopez-Rodriguez, A.B., Jimenez-Garcia, S., Sola-Carvajal, A., Garcia-Carmona, F. & Sanchez-Ferrer, A. Molecular characterization of a novel N-acetylneuraminate lyase from Lactobacillus plantarum WCFS1. *Appl. Environ. Microb.* **77**, 2471-2478 (2011).

9. Schauer, R. & Wember, M. Isolation and characterization of sialate lyase from pig kidney. *Biol. Chem. Hoppe Seyler* **377**, 293-9 (1996).

10. Barbosa, J.A.R.G., Smith, B.J., DeGori, R., Ooi, H.C., Marcuccio, S.M., Campi, E.M., Jackson, W.R., Brossmer, R., Sommer, M. & Lawrence, M.C. Active site modulation in the N-acetylneuraminate lyase sub-family as revealed by the structure of the inhibitor-complexed Haemophilus influenzae enzyme. *J. Mol. Biol.* **303**, 405-421 (2000).

11. Izard, T., Lawrence, M.C., Malby, R.L., Lilley, G.G. & Colman, P.M. The three-dimensional structure of N-acetylneuraminate lyase from Escherichia coli. *Structure* **2**, 361-9 (1994).

12. Lawrence, M.C., Barbosa, J.A.R.G., Smith, B.J., Hall, N.E., Pilling, P.A., Ooi, H.C. & Marcuccio, S.M. Structure and mechanism of a sub-family of enzymes related to N-acetylneuraminate lyase. *J. Mol. Biol.* **266**, 381-399 (1997).

13. Joerger, A.C., Mayer, S. & Fersht, A.R. Mimicking natural evolution in vitro: an N-acetylneuraminate lyase mutant with an increased dihydrodipicolinate synthase activity. *Proc. Natl. Acad. Sci. U. S. A.* **100**, 5694-9 (2003).
